# Genetics Visit Uptake Among Individuals Receiving Clinically Actionable Genomic Screening Results

**DOI:** 10.1001/jamanetworkopen.2024.2388

**Published:** 2024-03-15

**Authors:** Marci L. B. Schwartz, Whitney S. McDonald, Miranda L. G. Hallquist, Yirui Hu, Cara Z. McCormick, Nicole L. Walters, Jessica Tsun, Krista Zimmerman, Amie Decker, Celia Gray, Jennifer Malinowski, Amy C. Sturm, Adam H. Buchanan

**Affiliations:** 1 Department of Genomic Health, Geisinger, Danville, Pennsylvania; 2 Ted Rogers Centre for Heart Research, Cardiac Genome Clinic, Division of Clinical and Metabolic Genetics, The Hospital for Sick Children, Toronto, Ontario, Canada; 3 Department of Population Health Sciences, Geisinger, Danville, Pennsylvania; 4 University of Arkansas Medical Sciences, Little Rock; 5 Phenomics and Clinical Data Core, Geisinger, Danville, Pennsylvania; 6 23andMe, Sunnyvale, California

## Abstract

**Importance:**

Screening unselected populations for clinically actionable genetic disease risk can improve ascertainment and facilitate risk management. Genetics visits may encourage at-risk individuals to perform recommended management, but little has been reported on genetics visit completion or factors associated with completion in genomic screening programs.

**Objective:**

To identify factors associated with postdisclosure genetics visits in a genomic screening cohort.

**Design, Setting, and Participants:**

This was a cohort study of biobank data in a health care system in central Pennsylvania. Participants’ exome sequence data were reviewed for pathogenic or likely pathogenic (P/LP) results in all genes on the American College of Medical Genetics and Genomics Secondary Findings list. Clinically confirmed results were disclosed by phone and letter. Participants included adult MyCode biobank participants who received P/LP results between July 2015 and November 2019. Data were analyzed from May 2021 to March 2022.

**Exposure:**

Clinically confirmed P/LP result disclosed by phone or letter.

**Main Outcomes and Measures:**

Completion of genetics visit in which the result was discussed and variables associated with completion were assessed by electronic health record (EHR) review.

**Results:**

Among a total of 1160 participants (703 [60.6%] female; median [IQR] age, 57.0 [42.1-68.5] years), fewer than half of participants (551 of 1160 [47.5%]) completed a genetics visit. Younger age (odds ratio [OR] for age 18-40 years, 2.98; 95% CI, 1.40-6.53; OR for age 41-65 years, 2.36; 95% CI, 1.22-4.74; OR for age 66-80 years, 2.60; 95% CI, 1.41-4.98 vs age ≥81 years); female sex (OR, 1.49; 95% CI, 1.14-1.96); being married (OR, 1.74; 95% CI, 1.23-2.47) or divorced (OR, 1.80; 95% CI, 1.11-2.91); lower Charlson comorbidity index (OR for score of 0-2, 1.76; 95% CI, 1.16-2.68; OR for score of 3-4, 1.73; 95% CI, 1.18-2.54 vs score of ≥5); EHR patient portal use (OR, 1.42; 95% CI, 1.06-1.89); living closer to a genetics clinic (OR, 1.64; 95% CI, 1.14-2.36 for <8.9 miles vs >20.1 miles); successful results disclosure (OR for disclosure by genetic counselor, 16.32; 95% CI, 8.16-37.45; OR for disclosure by research assistant, 20.30; 95% CI, 10.25-46.31 vs unsuccessful phone disclosure); and having a hereditary cancer result (OR, 2.13; 95% CI, 1.28-3.58 vs other disease risk) were significantly associated with higher rates of genetics visit completion. Preference to follow up with primary care was the most common reported reason for declining a genetics visit (68 of 152 patients [44.7%]).

**Conclusions and Relevance:**

This cohort study of a biobank-based population genomic screening program suggests that targeted patient engagement, improving multidisciplinary coordination, and reducing barriers to follow-up care may be necessary for enhancing genetics visit uptake.

## Key Points

**Question:**

What factors are associated with completion of a genetics visit in a population with positive genomic screening results?

**Findings:**

In this cohort study of a population genomic screening program including 1160 participants, several demographic and program-level factors were associated with the likelihood of completing a follow-up genetics visit. Desire to follow-up with primary care was the most frequently reported reason for declining to schedule a genetics visit.

**Meaning:**

These findings suggest genomic screening programs may be more successful at supporting patients and clinicians in translating genetic results into clinical action by providing a framework for care coordination among primary care practitioners, genetics clinicians, and specialists.

## Teaser

This cohort study evaluates factors associated with follow-up genetics visits among individuals who received clinically actionable results of genomic screening.

## Introduction

Mining genomic data for clinically actionable monogenic findings enables identification of individuals with elevated disease risks, thus facilitating prevention, early detection, and treatment strategies.^1^ Genetic findings are considered clinically actionable when there is compelling evidence that they are associated with significant increases in disease risks and that there are interventions to mitigate these risks.^2,3,4^ Several sequenced cohorts built through research and institutional initiatives include disclosure of genomic results to consenting participants.^5,6,7,8,9,10,11^ Many of these programs interrogate genomic data for specific types of results across patients regardless of medical history, which can be referred to as genomic screening. Each program determines which genetic findings to report based on established lists of genes considered actionable in other contexts such as the US Centers for Disease Control and Prevention’s Tier 1 designation^9,12,13^ or the American College of Medical Genetics and Genomics’ secondary findings list.^14,15,16,17^

Genomic screening differs from standard clinical genetics models that rely on personal and/or family medical history as an indication for genetic testing.^18^ Thus, penetrance, expressivity, and age of onset may differ among individuals whose genetic results are ascertained through a genomic screening program instead of indication-based genetic testing.^19,20,21,22,23,24^ This underscores the need for additional research to determine the clinical utility of genomic screening, develop management guidelines for individuals identified through such screening, and determine whether genomic screening practices can lead to significant improvement in population health.^25^

For genomic screening to achieve anticipated disease prevention and early detection outcomes, identification of risk must be followed by patients and clinicians using genomic information to guide care. Completing a genetic counseling appointment has been associated with completing recommended risk management.^6,26,27^ Therefore, understanding factors associated with genetics visit completion in population genomic screening cohorts can inform future program design and help determine the clinical utility of returning genomic screening results. Here, we report on factors associated with completion of genetics visits and discuss implications for genomic screening program design.

## Methods

### Study Setting

This study was conducted under protocols approved by the Geisinger institutional review board; participants gave written informed consent. This report follows the Strengthening the Reporting of Observational Studies in Epidemiology (STROBE) reporting guideline. Geisinger is an integrated health system serving over 2 million individuals in central and northeastern Pennsylvania.^11,28^ Descriptions of Geisinger’s MyCode program and methods for disclosing genomic screening results have been published elsewhere.^11,16^ Briefly, program participants consent to donate blood and saliva samples to the biobank for health discovery research and to receive clinically actionable genomic results if identified. The informed consent form describes these results in broad terms (eg, increased risks for cancer but not for untreatable conditions such as Alzheimer disease) (eAppendix 1 in Supplement 1). Program participants cannot select specific types of genetic results to receive. Pathogenic or likely pathogenic (P/LP) variants in genes designated as actionable for adults by the American College of Medical Genetics and Genomics^14^ are identified by screening research-based exome sequence data through a variant filtering process that prioritizes variants associated with putative loss-of-function and/or variants on which multiple genetic testing laboratories have reached consensus on pathogenicity.^16^ Results are confirmed by sending a second, already available DNA sample from the biobank to a Clinical Laboratory Improvement Amendments–certified laboratory. Clinically confirmed P/LP variant results are disclosed to the participant and their primary care practitioner (PCP) and are documented in the electronic health record (EHR).^11^ PCPs receive a brief document that lists management guidelines, disease risks, instructions for referring to internal specialists for evaluation and management, and links to additional educational material (eg, from GeneReviews).

Three attempts are made by phone and/or secure EHR patient portal to reach program participants with their result. A standardized phone script is used that highlights the nature of the result, the importance of follow-up clinical care, and encourages communication with family members (eAppendix 2 in Supplement 1). A complimentary genetics visit is offered for participants to learn more about how the result could impact their and their relatives’ health. Genetics visits are scheduled with a genetic counselor (GC) and/or a medical geneticist when a diagnostic examination is indicated (eg, tuberous sclerosis); these genetics clinicians are experts in the relevant genetic conditions. Visits are available by phone, in office, or telehealth. Because the clinicians are supported by internal funding from the program, visits are typically available within 2 weeks of disclosure. All participants are sent a follow-up mailing with result-related information and details on scheduling a genetics visit (eAppendix 3 in Supplement 1). For those not reached for disclosure, this mailing is sent by certified mail. Participants who did not complete a genetics visit or who were not reached for disclosure were called 1 month postdisclosure to encourage visit completion.

### Data Collection: Demographics, Genetics Visits, and Procedures

Program participants who received a P/LP genomic screening result between July 2015 and November 2019 were included. Individuals were excluded if they had prior knowledge of the genetic result (typically because of previous clinical testing), had withdrawn from the program, were deceased before or within 60 days of the date the result was uploaded in their EHR, had a variant that was reclassified to uncertain significance, or were under age 18. Participants who received a result in the *HFE* gene were excluded, as there is a different disclosure workflow.

Participant characteristics and genetics visit details were collected in March and April 2021 through data pulled from the Geisinger EHR and the Genomic Screening and Counseling program’s Research Electronic Data Capture (REDCap) database version 10.6.9 (Vanderbilt University).^29,30^ EHR review was used for clarification and to identify missing data. A genetics visit was defined as any completed visit with a genetics clinician (GC and/or medical geneticist) within our health care system during which the program result was discussed. Genetics visits with the study team were assumed to have included results discussion. Medical record notes were reviewed for documentation of program result discussion for visits with other genetics clinicians in our health care system. For results disclosed in September 2019 or later, reasons for declining a genetics visit were collected via a standardized question on the disclosure phone script and recorded in the EHR. Additional medical record review was completed for the subset of individuals who received a result after implementing this change to gather reasons for declining visits and assess the association of the 1-month follow-up call with visit uptake in this group. Reasons for declining a visit were categorized by theme and double-coded by 2 authors (N.W. and M.L.B.S.); discrepancies were resolved by consensus.

### Statistical Analysis

Participants’ characteristics were summarized using descriptive statistics. Categorical variables were described using frequency with percentages and converted to dummy variables in a regression model (eTable 1 in Supplement 1). Continuous variables were described using medians and IQRs. Pearson χ^2^ and Wilcoxon rank-sums tests were used to compare differences between groups for categorical and continuous variables, respectively. The primary outcome was the completion of a genetics visit. Multivariable logistic regression models were used to evaluate the associations between participant characteristics and genetics visit completion. The following participant characteristics that could have been associated with genetics visit completion and were readily available in the EHR were included in the initial model (eTable 2 in Supplement 1): age at results disclosure in years, sex assigned at birth, self-reported race, marital status, employment status, Charlson comorbidity index,^31^ primary care physician status, EHR patient portal user, genetic result category, distance from home address to closest genetics clinic in miles, and time from results disclosure to data collection in years. Race was included in this study to determine whether there was a racial disparity in visit completion. Backward elimination with a mild significance level criterion of 0.20 was implemented for variable selection in the logistic regression model. Odds ratios (ORs) and 95% CIs were presented. Among the subset of participants with results disclosed between September and November 2019, the association between a 1-month follow-up call and genetics visit completion was examined. We calculated ORs and 95% CIs to compare successful vs unsuccessful 1-month follow-up call attempts. Statistical analyses were performed using RStudio version 2023.03.0 (R Project for Statistical Computing). *P* values of less than .05 were considered statistically significant. Data were analyzed from May 2021 to March 2022.

## Results

### Participant Characteristics

**Figure 1.**
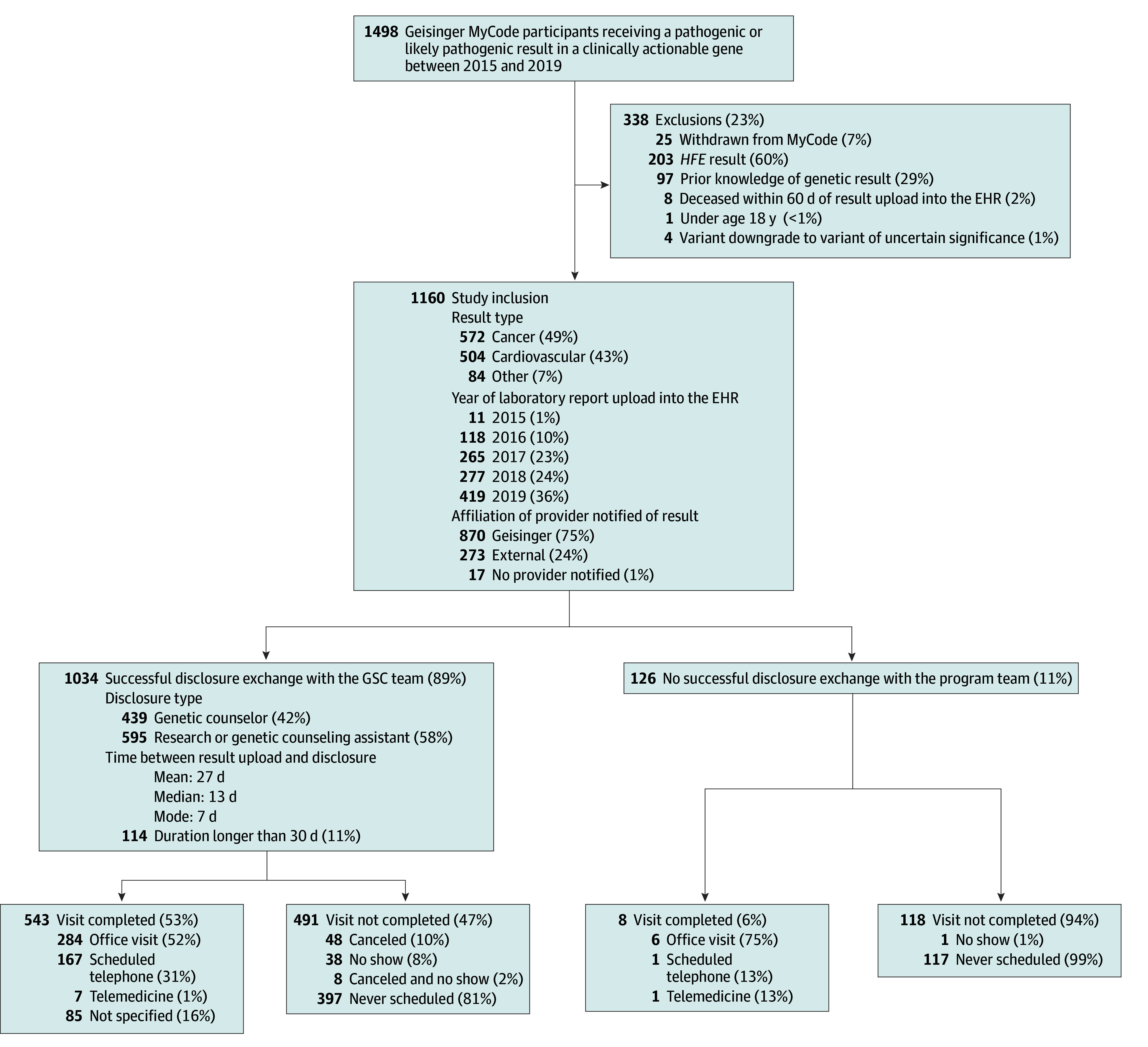
Inclusion Criteria, Result and Disclosure Details, and Visit Details for Program Participants EHR indicates electronic health record; GSC, Geisinger Commonwealth School of Medicine; HFE, hereditary hemochromatosis.

Characteristics of the 1160 participants who met inclusion criteria are summarized in Table 1. A total of 703 participants were female (60.6%), and the median (IQR) age was 57.0 years (42.1-68.5) years. Median (IQR) time between result upload to the EHR and data collection was 2.7 (1.5-3.7) years. Most participants received results for hereditary cancer predisposition risk (92 of 1160 participants [49.3%]) or hereditary cardiovascular disease risk (504 of 1160 participants [43.4%]) (Figure 1, Table 1; eTable 3 in Supplement 1). Most participants, 1034 of 1160 (89%), had a successful result disclosure discussion with the study team before data collection (Figure 1). Forty-two percent (439 of 1034) of successful disclosures were made by a genetic counselor; the remainder were made by a research or genetic counseling assistant (RA/GCA) (Figure 1).

**Table 1.** Participant Characteristics and Genetics Visit Completion Rates

	Patients, No. (%)	
Variable	Total	Completed genetics visit
Study sample	1160	551 (47.5)
Age at disclosure, y		
18-40	259 (22.3)	138 (53.3)
41-65	524 (45.2)	256 (48.9)
66-80	294 (25.3)	140 (47.6)
≥81	83 (7.2)	17 (20.5)
Sex		
Female	703 (60.6)	361 (51.4)
Male	457 (39.4)	190 (41.6)
Race		
Asian, Black or African American, Native Hawaiian or other Pacific Islander, or unknown^a^	28 (2.4)	14 (50.0)
White	1132 (97.6)	537 (47.4)
Marital status		
Divorced/separated	148 (12.8)	71 (48.0)
Married/significant other	666 (57.4)	346 (52.0)
Single	236 (20.3)	98 (41.5)
Widowed	109 (9.4)	36 (33.0)
Missing	1 (0.1)	0
Employment status		
Working	479 (41.3)	260 (54.3)
Not working	290 (25.0)	128 (44.1)
Retired	390 (33.6)	163 (41.8)
Missing	1 (0.1)	0
Charlson comorbidity index		
0-2	590 (50.9)	306 (51.9)
3-4	255 (22.0)	131 (51.4)
≥5	315 (27.2)	114 (36.2)
Primary care practitioner status		
Internal	870 (75.0)	428 (49.2)
External	290 (25.0)	123 (42.4)
Patient portal user		
No	353 (30.4)	136 (38.5)
Yes	807 (69.6)	415 (51.4)
Gene category		
Cancer risk^b^	572 (49.3)	289 (50.5)
Cardiovascular disease risk^c^	504 (43.4)	230 (45.6)
Other disease risk^d^	84 (7.2)	32 (38.1)
Disclosure staff type		
Genetic counselor	439 (37.8)	217 (49.4)
Research assistant	595 (51.3)	326 (54.8)
Not disclosed	126 (10.9)	8 (6.3)
Distance to genetics clinic in miles		
0-8.9	294 (25.3)	154 (52.4)
8.9-13.3	312 (26.9)	151 (48.4)
13.3-20.1	278 (24.0)	128 (46.0)
>20.1	276 (23.8)	118 (42.8)
Follow-up time, y		
<2	398 (34.3)	167 (42.0)
2-3	289 (24.9)	141 (48.8)
3-4	240 (20.7)	119 (49.6)
4-5	160 (13.8)	86 (53.8)
>5	73 (6.3)	38 (52.1)

^a^ Asian (n = 5), Black or African American (n = 20), Native Hawaiian or other Pacific islander (n = 2), unknown (n = 1). These racial categories were grouped together due to small numbers in each individual category.

^b^ Hereditary breast or ovarian cancer syndrome (*BRCA1*, *BRCA2* genes), Lynch syndrome (*MLH1*, *MSH2*, *MSH6*, *PMS2* genes), multiple endocrine neoplasia type 1 (*MEN1* gene), multiple endocrine neoplasia type 2 (*RET* gene), hereditary paraganglioma-pheochromocytoma syndrome (SD*HAF2*, SD*HB*, SD*HC*, SD*HD* genes), von Hippel-Lindau syndrome (*VHL* gene), familial adenomatous polyposis (*APC* gene), *PTEN* hamartoma tumor syndrome (*PTEN* gene), hereditary retinoblastoma (*RB1* gene), Li-Fraumeni syndrome (*TP53* gene), tuberous sclerosis complex (*TSC1*, *TSC2* genes).

^c^ Long QT syndrome or Brugada syndrome (*KCNH2*, *KCNQ1*, *KCNE1*, *SCN5A* genes), arrhythmogenic cardiomyopathy (*DSC2*, *DSG2*, *DSP*, *PKP2* genes), dilated or hypertrophic cardiomyopathy (*LMNA*, *MYBPC3*, *MYH7*, *MYL2*, *TNNI3*, *TNNT2*, *TPM1* genes), Fabry disease (*GLA* gene), familial hypercholesterolemia (*LDLR*, *APOB* genes), hereditary thoracic aortic disease (*ACTA2* gene), Marfan syndrome (*FBN1* gene), Loeys-Dietz syndrome (*SMAD3*, *TGFBR1* genes), vascular Ehlers-Danlos syndrome (*COL3A1* gene), hereditary hemorrhagic telangiectasia (*ENG* gene).

^d^ Malignant hyperthermia susceptibility (*RYR1* gene), dual result (*BRCA1* + *LDLR*, *LMNA + SCN5A*, *BRCA2 + LDLR, ACTA2 + BRCA1, APOB + BRCA2, BRCA2 + DSP, BRCA2 + PKP2*).

### Visit Completion

**Figure 2.**
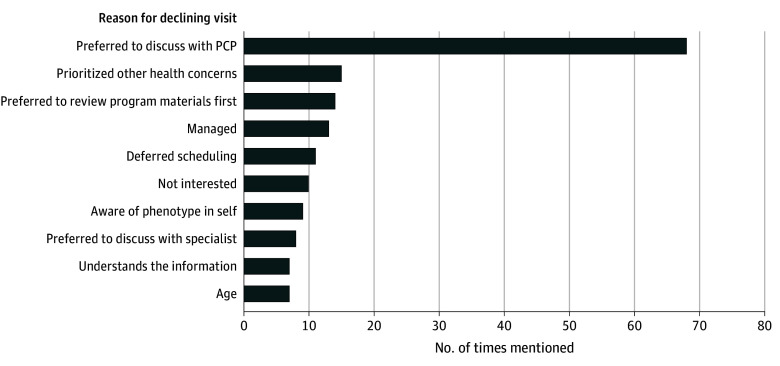
Participant-Cited Reasons for Declining a Genetics Visit The 10 most frequently cited reasons are shown in the graph (number of times cited, % of participants citing). A total of 200 reasons were mentioned among 23 categories from 152 unique participants, 21 of whom ultimately completed a visit. Age was primarily cited among participants with advanced age. “Managed” references a participant’s perception that they were being managed for the condition and/or the phenotype(s) associated with a condition. PCP indicates primary care practitioner.

Fewer than half of participants in the full cohort, 551 of 1160 (47.5%), completed a genetics visit (Figure 1). For participants who did not complete a visit, the majority (514 of 609 participants [84.4%]) never scheduled a genetics visit. In the fall 2019 cohort, the most common documented reason for declining a genetics visit was a preference to follow-up with a PCP (68 of 152 participants [44.7%]) (Figure 2). Other less frequent reasons cited by participants include wanted to discuss with a family member first (6 participants [4.0%]); busy or time obligation (6 participants [4.0%]); already discussed with another health care practitioner (6 participants [4.0%]); aware of family history of phenotype or result (5 participants [3.3%]); lives out of state (4 participants [2.6%]); refused to provide a reason (2 participants [1.3%]); cost (2 participants [1.3%]); prioritized family member’s health concerns (2 participants [1.3%]); anxious or worried (1 participant [0.7%]); future genetics appointment scheduled (1 participant [0.7%]), overwhelmed (1 participant [0.7%]); scheduling conflicts (1 participant [0.7%]); and transferring care out of system due to insurance (1 participant [0.7%]). No reason was collected for 17 participants.

### Factors Associated With Genetics Visit Completion

Completion rates of visits for each categorical variable are shown in Table 1. Race, primary care physician status, employment status, and time from results disclosure to data collection were removed from the final multivariable logistic regression model (eTable 2 and eTable 4 in Supplement 1). Significant associations from the final model are presented in Table 2; all associations are in eTable 5 and eTable 6 in Supplement 1. Younger age (ages 18-40 OR, 2.98; 95% CI, 1.40-6.53; ages 41-65 OR, 2.36; 95% CI, 1.22-4.74; and ages 66-80 OR, 2.60; 95% CI, 1.41-4.98 compared with age ≥81) was associated with higher visit completion rates. Female participants were more likely to complete a visit than male participants (51.4% vs 41.6%; OR, 1.49; 95% CI, 1.14-1.96; *P* = .004). Lower comorbidity index score was associated with higher completion rates (score of 0-2: 51.9% vs 36.2%; OR, 1.76; 95% CI, 1.16-2.68; *P* = .01; score of 3-4: 51.4% vs 36.2%; OR, 1.73, 95% CI, 1.18-2.54; *P* = .01 compared with score of ≥5). Married and divorced participants were more likely than single participants to complete a visit (married: 52.0% vs 41.5%; OR, 1.74; 95% CI, 1.23-2.47; *P* = .002; divorced: 48.0% vs 41.5%; OR, 1.80; 95% CI, 1.11-2.91; *P* = .02). Participants who used the EHR patient portal were more likely to complete a visit (51.4% vs 38.5%, OR, 1.42; 95% CI, 1.06-1.89; *P* = .02), as were participants who lived closer to a genetics clinic (OR, 1.64; 95% CI, 1.14-2.36 for <8.9 miles vs >20.1 miles). Participants with hereditary cancer risk had higher completion rates than those with other disease risk (50.5% vs 38.1%; OR, 2.13; 95% CI, 1.28-3.58; *P* = .004). Participants whose results were disclosed by a GC or RA/GCA had significantly higher completion rates than those who could not be reached for disclosure (GC: 49.4% vs 6.3%; OR, 16.32; 95% CI, 8.16-37.45; *P* < .001; RA: 54.8% vs 6.3%; OR, 20.30; 95% CI, 10.25-46.31; *P* < .001). There was no difference in visit completion for participants whose result was disclosed by a GC vs a RA/GCA (eTable 7 in Supplement 1). On analysis of the subpopulation who were successfully reached for disclosure, there was no change in which variables were significantly associated with genetics visit completion (eTable 7 in Supplement 1).

**Table 2.** Factors Significantly Associated With Completion of Genetics Visit

Variable	OR (95% CI)	*P* value
Age, y		
18-40	2.98 (1.40-6.53)	.01
41-65	2.36 (1.22-4.74)	.01
66-80	2.60 (1.41-4.98)	.003
≥81	1 [Reference]	NA
Sex		
Female	1.49 (1.14-1.96)	.004
Male	1 [Reference]	NA
Marital status		
Single	1 [Reference]	NA
Married/significant other	1.74 (1.23-2.47)	.002
Divorced	1.80 (1.11-2.91)	.02
Charlson comorbidity index		
0-2	1.76 (1.16-2.68)	.01
3-4	1.73 (1.18-2.54)	.01
≥5	1 [Reference]	NA
Patient portal use		
Yes	1.42 (1.06-1.89)	.02
No	1 [Reference]	NA
Gene category		
Cancer	2.13 (1.28-3.58)	.004
Other	1 [Reference]	NA
Disclosure		
Disclosed by GC	16.32 (8.16-37.45)	<.001
Disclosed by RA	20.30 (10.25-46.31)	<.001
Not disclosed	1 [Reference]	NA
Distance to clinic, mi		
0-8.9	1.64 (1.14-2.36)	.01
>20.1	1 [Reference]	NA

Abbreviations: GC, genetic counselor; NA, not applicable; RA, research assistant.

Data from the fall 2019 cohort were used to assess the association between a 1-month follow-up call and visit completion. This call was significantly associated with visit completion status among those who had not previously been reached for disclosure (OR, 3.56; 95% CI, 1.06-11.91; *P* = .047) but not among those who had previously been reached by the team and had declined scheduling a genetics visit (OR, 1.30; 95% CI, 0.45-3.83; *P* = .64) (eTable 8 in Supplement 1).

## Discussion

Effective disease prevention and early detection prompted by genomic screening relies on patients and clinicians using genomic information to guide surveillance recommendations and medical management. Prior work on a subset of this cohort revealed a significant association between genetics visit completion and performance of management recommendations.^26^ This association may be related to care coordination within the health system, as a primary goal of genetics visits in this context is to facilitate risk-related follow-up through patient education and psychosocial support as well as referral to disease-specific specialists. Genomic screening programs that incorporate genetics visits after results disclosure may, therefore, bolster risk-related management. This study sought to identify factors associated with genetics visit completion in hopes of guiding genomic screening programs in facilitating follow-up care for patients with newly identified genetic risk. Lessons learned from this program could be applicable to other population genomics initiatives, such as the National Institutes of Health *All of Us* research program.

Notably, fewer than half of participants completed a complimentary genetics visit. The most common reason participants cited for declining a genetics visit was that they preferred to follow-up with their PCP. This highlights an area where additional support of clinicians and patients may facilitate result-related management. A qualitative interview study of PCPs involved with another genomic screening program found that PCPs recognized the value of genetic testing in risk stratification and disease prevention but identified barriers related to how to prioritize the information among other preventive care, and a perceived lack of knowledge and skill related to managing positive results.^32^ To facilitate downstream care, genomic screening programs should prioritize supporting PCPs in providing appropriate medical management and facilitating referrals to specialty care. This underscores that access to genetics and other specialty care, which vary widely by region in the US,^33,34,35^ must be emphasized by genomic screening programs that seek to encourage recommended risk management. The importance of improving access to care was also apparent in the association we found between greater distance to genetics clinics and lesser completion of genetics visits.

We identified several variables to consider when designing genomic screening programs. The association between reaching a participant for phone disclosure of results and visit completion suggests that a conversation with at-risk individuals is critical to encouraging a genetics visit. For genomic screening programs based in research programs, as in *All of Us*,^15^ MyCode,^11^ and several other health system-based programs,^9,13,36^ these conversations may be particularly salient in helping individuals navigate potential challenges in the transition from being a research participant to becoming a patient identified to be at increased risk for cancer, cardiovascular disease, or other serious diseases. Additionally, the finding that, when following a phone script, RA/GCAs were as effective at encouraging completion of a genetics visit as GCs could help programs determine how to allocate effort among genetic counselors and GCAs. Furthermore, we found that follow-up phone calls were effective at promoting a genetics visit only among participants who had not previously been reached for a disclosure call. This suggests that focusing these calls on unreached individuals rather than on those who had previously declined a genetics visit may be an efficient allocation of resources.

Findings also highlight groups that may need additional support in understanding results’ implications for themselves and their relatives and in incorporating results into their care. Lower rates of genetics visit completion among participants over age 80 and among those with higher comorbidity scores could indicate that these individuals perceive the genetic result as less impactful for their care. Further research that elucidates their preferences and needs is indicated. Such research could also explore why participants with a genetic result not associated with cancer or cardiovascular disease (which were primarily malignant hyperthermia susceptibility), single individuals, and males were less likely to have a genetics visit.

Finally, this study suggests that patients’ engagement in their care may be an important factor in visit completion, as patients who were active in the EHR portal showed higher visit completion rates. Such portals may be an important patient engagement tool, especially when they include features that promote visit completion such as reminders and the ability to view upcoming appointment details.^37,38,39^ Identifying ways to actively engage patients in the management of their genomic screening results may support completion of recommended follow-up care.

### Limitations

An important limitation of this study is that genetics visit uptake data were restricted to those performed within the Geisinger system. It is unclear to what extent individuals may have had genetics care outside of Geisinger. However, Geisinger is a rural, integrated health system that serves a relatively stable population with limited local options for alternative genetics services. Additionally, because study participants lacked racial and ethnic diversity, the study may not capture factors of importance in more racially and ethnically diverse cohorts. While we reached nearly 90% of participants to disclose their genetic results, it is possible that some of those who received the results mailing only did not glean the medical importance of a genetics visit due to limited health or genomic literacy. Furthermore, it is possible that outstanding questions regarding penetrance and expressivity in the genomic screening context influenced visit completion. Due to using data readily available in the EHR, this study was not designed to investigate the potential impact of this uncertainty on genetics visit uptake. Finally, participants provided informed consent to participate in a large precision health project, with no specific primary indication for genetic testing identified at that time. Findings may be less applicable to programs with extensive consent procedures that select for highly engaged participants or groups with an a priori medical indication for testing.

## Conclusions

This study highlights the need for genomic screening programs to support patients and clinicians in translating genetic results into clinical action to benefit from disease prevention and early detection. Strategies that promote patient engagement through an initial discussion about their result and those that reduce barriers to additional downstream care may be effective. Providing a framework for care coordination among PCPs, genetics clinicians, and specialists may pose challenges to screening programs not embedded in health care systems.

## Notes

Supplement 1. **eAppendix 1. **MyCode Community Health Initiative Informed Consent Form **eAppendix 2. **Phone Script: Disclosure of a MyCode Result **eAppendix 3. **Patient Information Packet Example Documents Used During Study Time Frame **eTable 1. **Dummy Coding for Categorical Variables **eTable 2. **Variable Selection Before and After Backward Elimination **eTable 3. **Number of Participants With Results by Gene **eTable 4. **Visit Completion (n, %) as Stratified by Follow-up Duration in Years Since Result Disclosure **eTable 5. **Association of Participant Characteristics With Visit Completion **eTable 6. **Participant Characteristics Stratified by Genetics Visit Completion, Only Including Variables Selected for Logistic Regression **eTable 7. **Factors Significantly Associated With Completion of Genetics Visit Among Those Reached for Disclosure **eTable 8.** Relationship Between 1-Month Follow-up Calls and Visit Completion Status Among Those Who Did Not Schedule a Visit During an Initial Disclosure Attempt

Supplement 2.
Data Sharing Statement
