# Supplementary material for: Genetics Visit Uptake Among Individuals Receiving Clinically Actionable Genomic Screening Results
Source: JAMA Netw Open. 2024 Mar 15;7(3):e242388. doi: 10.1001/jamanetworkopen.2024.2388 (PMC10943406; doi:10.1001/jamanetworkopen.2024.2388)
Supplement: Supplement 1. — eAppendix 1. MyCode Community Health Initiative Informed Consent Form eAppendix 2. Phone Script: Disclosure of a MyCode Result eAppendix 3. Patient Information Packet Example Documents Used During Study Time Frame eTable 1. Dummy Coding for Categorical Variables eTable 2. Variable Selection Before and After Backward Elimination eTable 3. Number of Participants With Results by Gene eTable 4. Visit Completion (n, %) as Stratified by Follow-up Duration in Years Since Result Disclosure eTable 5. Association of Participant Characteristics With Visit Completion eTable 6. Participant Characteristics Stratified by Genetics Visit Completion, Only Including Variables Selected for Logistic Regression eTable 7. Factors Significantly Associated With Completion of Genetics Visit Among Those Reached for Disclosure eTable 8. Relationship Between 1-Month Follow-up Calls and Visit Completion Status Among Those Who Did Not Schedule a Visit During an Initial Disclosure Attempt [file jamanetwopen-e242388-s001.pdf]

Supplemental Online Content

Schwartz MLB, McDonald WS, Hallquist MLG, et al. Genetics visit uptake among individuals receiving clinically actionable genomic screening results. *JAMA Netw Open*. 2024;7(3):e242388. doi:10.1001/jamanetworkopen.2024.2388

eAppendix 1. MyCode Community Health Initiative Informed Consent Form

eAppendix 2. Phone Script: Disclosure of a MyCode Result

eAppendix 3. Patient Information Packet Example Documents Used During Study Time Frame

eTable 1. Dummy Coding for Categorical Variables

eTable 2. Variable Selection Before and After Backward Elimination

eTable 3: Number of Participants With Results by Gene

eTable 4. Visit Completion (n, %) as Stratified by Follow-up Duration in Years Since Result Disclosure

eTable 5. Association of Participant Characteristics With Visit Completion

eTable 6. Participant Characteristics Stratified by Genetics Visit Completion, Only Including Variables Selection for Logistic Regression

eTable 7. Factors Significantly Associated With Completion of Genetics Visit Among Those Reached for Disclosure

eTable 8. Relationship Between 1-Month Follow-up Calls and Visit Completion Status Among Those who did not Schedule a Visit During an Initial Disclosure Attempt

This supplemental material has been provided by the authors to give readers additional information about their work.

© 2024 Schwartz MLB et al. *JAMA Network Open.*

eAppendix 1. MyCode Community Health Initiative Informed Consent Form

RESEARCH CONSENT/AUTHORIZATION FORM
GEISINGER MYCODE PROJECT
PROJECT 2006-0258

TITLE OF STUDY: Geisinger MyCode Project
PRINCIPAL INVESTIGATOR: David J. Carey, PhD
PHONE NUMBER: 1-866-910-6486 (toll free)
24-HOUR PHONE NUMBER: 570-271-6211 (HOSPITAL SWITCHBOARD)

You are invited to take part in a project, called Geisinger MyCode. This project involves collecting blood, saliva, and health information from thousands of people for use in future research studies. Please read this information carefully before making your decision whether or not to take part.

THIS FORM SERVES TWO PURPOSES:

1) Provides information about the project and the possible benefits and risks involved.

2) Describes protected health information (PHI) that will be obtained during the project. It describes how the PHI will be used and with whom it will be shared.

This project is being conducted by Geisinger Clinic. Funds to support the collection and storage of samples may come from other external sources.

WHY DO YOU WANT TO STORE MY BLOOD AND SALIVA SAMPLES AND HEALTH INFORMATION?

Researchers usually study diseases in patients after the disease develops. We would like to store samples of your blood and saliva for future use so that researchers can study your genes and other components in your blood (for example, proteins). A sample of your saliva can also be used to study your genes. Your samples contain genes, which are made up of DNA and which serve as the "instruction book" for the cells that make up our bodies. Researchers may study your genes (instruction book) in many ways. They may focus on a single gene, multiple genes, or all of your genes. Studying all of your genes is sometimes referred to as whole exome or whole genome sequencing. We may also use other methods as they are developed.

This information about your genes, along with health information from your electronic medical record may help researchers understand the causes of diseases and how to treat or prevent them. This information may also be helpful to you and your doctor in guiding your medical care.

WILL YOU SHARE MY SAMPLES AND INFORMATION TO PROMOTE HUMAN HEALTH?

Yes, a portion of your sample and your health information might be shared with other researchers, including government agencies such as the National Institutes of Health (NIH) and other public and commercial partners. We will also share your genetic information (your unique sequence) with the researchers. The other researchers who study your samples, genetic information and health information will not know who you are because the samples, genetic information and health information will be protected through use of a special code known only to the Geisinger team. The other researchers will

© 2024 Schwartz MLB et al. *JAMA Network Open.*

access your genetic information but will not have access to information that is typically used to identify you. All research partners who have access to your information will be required by the Geisinger team to promise not to try to identify or contact you. We will not notify you every time your sample, genetic information or health information will be used.

HOW MANY PEOPLE WILL TAKE PART IN THIS PROJECT?
Our goal is to enroll the majority of Geisinger patients (over 500,000) in the project.

WHAT WILL I BE ASKED TO DO?
To participate in this project, every time you have blood drawn on your doctor's order, we may collect extra blood (up to about two tablespoons) for research. You may also choose to give a blood or saliva sample when you sign up or at other times during the project.

As part of this project we will look at your electronic medical record from time to time to update the information for this project and to collect information for future studies. This will take place for as long as your sample is stored, which may be many years unless you tell us to stop.

In the future there may be times when approved MyCode researchers need additional information. If this situation arises and you have agreed to be contacted, you will only be contacted by a member of the Geisinger team. You can decide if you want to be contacted about participation in any other approved studies or to provide additional information by checking the "Yes" or "No" box at the end of this form.

HOW LONG WILL I BE IN THE PROJECT?
If you decide to take part in the Geisinger MyCode project, your samples and health information will be stored. There is no plan to stop storing samples at any given point in the future, so the endpoint for and health information can be kept and used for, you can change your mind at any time.

ARE THERE BENEFITS TO TAKING PART IN THE PROJECT?
Research involving your samples and information may contribute to improvements in health care. This information may be important for the development of new tests and treatments.

You may or may not receive direct benefits from your participation in this project. In analyzing your genetic material, researchers may find information that could be specifically important to your healthcare, or to the healthcare of some of your family members. The types of information could include your risk for some cancers or your risk for an irregular heartbeat; or, if you need medication now or in the future, the information could help health care professionals be more precise with medication dosage.

At this time, we will **not** analyze or return any genetic information concerning untreatable conditions, including neurodegenerative diseases such as Alzheimer's Disease, Amyotrophic Lateral Sclerosis (ALS), Multiple Sclerosis, etc. As doctors and researchers learn more about genes, health and disease, the possibility of finding information important to your health care will increase.

© 2024 Schwartz MLB et al. *JAMA Network Open.*

If we find information that is determined to be important to your health care, we may share that information with both you and your doctor and place it in your electronic medical record. To help you and your doctor understand and use this information, the study team will provide educational materials about each result returned. Genetic counselors and other experts will work with your physician to return the results to you and will be available for any questions you may have. As part of this study, Geisinger will establish an Oversight Committee of physicians and other healthcare providers to decide *which* results should be returned to you and *how* those results will be returned to you. Research results will be validated in a clinical laboratory before being shared with you and your physician.

WHAT ARE THE RISKS?

If you are having blood drawn as a result of a doctor's order, drawing two extra tablespoons of blood for use by MyCode will not entail any additional risks to you. If you choose to give a blood sample at other times in the project, there is a small risk of bleeding, bruising, or infection at the needle site. Fainting during blood drawing is also rare but possible.

People who are specially trained to draw blood will obtain your samples for MyCode. Sterile needles will be used and other precautions taken to minimize these risks. There are no risks to giving a saliva sample.

If information important to your health care is found, it could surprise or upset you. We will have resources available, including genetic counselors and doctors, to help and support you.

The biggest risk to you in this project is the potential for an unexpected release of your genetic or protected health information, despite the best efforts of the Geisinger team to keep your information secure. Although no one can guarantee absolute confidentiality, or that your identity will never become known, several steps will be taken to protect your privacy to the best of our ability. The blood sample, saliva sample, and the health information will each be assigned a special code which could be linked back to you. To create this special code we will access identifiable information about you, known as protected health information ("PHI"). However, this code which links you and your blood, saliva, and health information will be strictly protected. Only a select number of people at Geisinger will have access to the code, all of whom will have received training in protecting privacy. The other researchers who study your information will not have access to this special code and will be required to promise not to try to identify you.

There also may be other privacy risks that we have not foreseen.

There is a federal law called Genetic Information Nondiscrimination Act (GINA). In general, this law makes it illegal for health insurance companies, group health plans, and most employers to discriminate against you based on your genetic information. Although this law does not protect against discrimination by companies that sell life, disability, or long-term care insurance, the chances that your information would be released to these companies are very small.

WHO ELSE MAY ACCESS MY PHI?

Your PHI may be disclosed if required by law. Regulatory organizations may inspect and/or copy your research records (including information in your hospital medical record) for quality assurance and data analysis. Those organizations include groups such as the Food and Drug Administration and

© 2024 Schwartz MLB et al. *JAMA Network Open.*

Department of Health and Human Services. Federal Privacy Regulations provide safeguards for privacy, security, and authorized access.

Geisinger Clinic has several departments that are responsible for making sure research is performed according to federal and state regulations. The staff members of these departments may review your medical record and research data for this study. This review will be administrative in nature and no PHI will be sent outside Geisinger Clinic for this purpose.

Once your PHI has been disclosed outside of Geisinger Clinic, the information may no longer be protected by federal privacy laws. Persons and organizations that receive your PHI might be able to disclose it to others without your permission.

WHAT ARE THE COSTS?
There will be no extra cost to you or your insurance company for taking part in the MyCode Project.

WHAT ARE MY RIGHTS AS A PARTICIPANT?
Taking part in this project is voluntary. You may choose not to be in the project or withdraw at any time. Your decision not to take part in or to withdraw from the project will not involve any penalty or loss of benefits. It will not affect your access to treatment or health care at any Geisinger health care facility. If you decide to withdraw from the project or to withdraw permission to collect your health information, we ask that you contact the study doctor, Dr. David Carey, 100 North Academy Avenue, Danville, PA 17822-26-30 in writing and let him know that you are withdrawing from the project. Even if you withdraw from the project, we may still use the information about you that we collected before you left the project. However, we will not use your samples for future research. No further samples or health information about you will be collected. We will not contact you for any future studies using this data.

Research results might someday lead to the development of a medical or genetic test, drug, or other commercial product. You will not have any ownership rights in such product(s) or the samples or information you contribute, and you will not receive money or any other form of payment for taking part in this project or from the sale of any such product(s).

WHOM DO I CALL IF I HAVE QUESTIONS OR PROBLEMS?
For questions about the project, contact a member of the project staff who can discuss the entire project plan with you and provide more information by calling toll free at 1-866-910-6486(press 2).

For questions about your rights as a research participant, please contact the Human Research Protection Program staff of the Geisinger Institutional Review Board at (570)-271-8663. When calling please reference study number 2006-0258.

WHERE CAN I GET MORE INFORMATION?
Make sure you read all the information in this form. You should keep this form. There are phone numbers in the beginning of this form you can call. Upon your request the project investigator can discuss the entire project plan with you and provide more information.

This consent does not have an expiration (ending) date.

© 2024 Schwartz MLB et al. *JAMA Network Open.*

Geisinger MyCode
Signature for Voluntary Consent/Authorization

Each time I have a blood draw, a blood sample may be collected and used for MyCode research. A blood or saliva sample may also be collected at other times in the project and used for MyCode Research. If we find information which we determine to be important to your health care, we may share that information with you and your doctor and place it in your electronic medical record.

Researchers may contact me to ask if I want to participate in future MyCode approved studies and to collect additional information:

[ ] Yes [ ] No

SIGNATURE

I have read the entire consent document entitled "Geisinger MyCode Project:
Research Consent and Authorization Form" version 20 dated 10/11/2013

I agree to take part in this research project.

____
Research Participant Name (Please Print)

____________________________________ ____________________________
Research Participant Signature Date

____________________________________ ____________________________
Signature of Legally Authorized Representative (LAR) Date
for **ADULTS NOT CAPABLE of GIVING CONSENT**

I confirm that I have explained the study to the participant and that the participant has agreed to be in the study.

____________________________________ ____________________________
Person Obtaining Consent Signature Date

© 2024 Schwartz MLB et al. *JAMA Network Open.*

eAppendix 2. Phone Script: Disclosure of a MyCode Result

1. **Connect:** Good morning/afternoon!
2. **Introduce:** This is "______ from Geisinger"
3. Ask if the patient is available; once patient is on the phone, reintroduce myself as "____________ from Geisinger's MyCode project."

4. **Communicate:** Ask if they remember the MyCode study that they signed up for ______, where they were asked to donate extra blood next time they went for labs, so that we can better understand genetics. Remind them that at that time we told you that we would contact you if we found anything that may be important to your health in your sample.
5. **Confirm patient by asking date of birth**
6. We did find a change in your DNA that is important for you to know about. We recently sent you a letter/MyGeisinger message about it.

7. **Ask:** Would now be a good time to talk about your results from the study?

8. Have you had any contact with your primary care provider about results from the study?

9. If **yes**, "great. Did they discuss the result with you?"
10. If **no,** say that is ok. We will let your PCP know of your result. Would you mind if I told you about your results and how your doctor might use them to take care of you?

11. **Respond:** Your testing found a gene change that increases your risk for ______________________________.

    a. **Female Hereditary Breast and Ovarian Cancer (*BRCA1/2*) example: "certain cancers such as breast cancer and ovarian cancer"**
    b. **Cardiomyopathy (*MYBPC3* and *MYH7*) example: "important heart problem like cardiomyopathy"**
    c. **Malignant Hyperthermia (*RYR1*) example: "a severe reaction to anesthesia such as high fever and muscle problems"**

12. This result does not mean that you _________. It does mean that you may have a higher chance than most people of getting it at some point in your life.

    a. **Female Hereditary Breast and Ovarian Cancer (*BRCA1/2*) example: "have cancer"**
    b. **Cardiomyopathy (*MYBPC3* and *MYH7*) example: "have a heart muscle weakness"**
    c. **Malignant Hyperthermia (*RYR1*) example: are certain to experience these problems when exposed to anesthesia. {**replace next sentence} It does mean that you have a higher chance than most**

© 2024 Schwartz MLB et al. *JAMA Network Open.*

people of having complications if exposed to these anesthesia medications, even if you have not had problems in the past”

13. We are calling because there are steps that can be taken to treat or prevent health issues related to this gene change. **There are three important recommendations: (a) see a health care provider about this result, (b) discuss this result with your family members, and (c) gather information on whether any of your family members have (heart problems, cancer, etc).**

14. ***The first recommendation* is that it is important that you meet with a health care provider who can help you understand this DNA change better and determine what to do next.**

    We would be happy to schedule a free visit with our Clinical Genomics team. That would involve meeting with a genetic counselor and other providers to talk about this condition and how it can be passed through families. They will answer any questions that you may have at that time in detail. They are very knowledgeable about _______________{condition name}.

    We see patients at our clinics in Danville/Bloomsburg/Forty Fort/State College. We also offer telephone appointments and secure videoconference visits to your home computer or smart phone. Would you be interested in setting up a free appointment? (if no go to 14)

15. You may also choose to meet with your primary care provider if you like.

16. At this time, do you know who you want to see about this result?

    a. If patients say that they are being treated currently: say that “we understand that you are being treated, which is great, however, knowing that this is a genetic condition can sometimes lead to changes in screening or treatment recommendations. We also want to obtain your family history so we can see how it may affect your family members as well. Your family can be screened and treated early for these issues so they can be as healthy as possible.”
    b. **If no:** That’s fine, we’d be happy to schedule you with Clinical Genomics at any time in the future if you decide. As a reminder, you can also follow up with your PCP or specialist who is familiar with this condition.
        1. Ask the patient if they would mind giving us an idea of why they are not interested…is it for any of the following reasons?
            a. *Aware of variant/finding and being managed*

© 2024 Schwartz MLB et al. *JAMA Network Open.*

b. *Aware of condition (not variant) and being managed*
c. *Too "old"*
d. *Not interested*
e. *Difficulty with getting here (cost, travel, etc)*
f. *Timing*
g. *Other*

17. **Understanding your family history is essential. Therefore, *we recommend* that you gather more information on your family.** You may need to talk with your family members to learn more about your family health history. Some examples of important information include, whether any family members have (heart problems, cancer etc.) and approximate ages such as the age they are now and when they were diagnosed. [(If scheduled with Clinical Genomics) You will be asked about your family history during your appointment.]

18. Family history is also important because it can help us determine which family members may also have a higher chance of developing ___________ {condition risk, e.g., heart problems, cancer}. Your children, siblings, parents, and other relatives that share genes with you may be at higher risk. This risk can be different in different family members, so it is very important to get this information to all of them [Alter for different inheritance patterns as needed].

19. **We recommend that you discuss this result with your relatives so that they can pursue the proper care and testing if they wish.** Because this is so important, our team has developed multiple tools to help you share your results with your relatives. One is an online tool, and the other is a letter you can mail or hand to your family. The link to access and use the online tool can be opened on your computer, tablet or mobile phone. Would you like us to send you the online tool? (Document yes/no in EPIC)
    a. If **yes –** Great! We can send it to your mobile phone (it has to be a smart phone) and/or to your email address (if they choose email, verify the email address in EPIC, and if not, enter it)

20. We will be mailing you a packet that will also include a family letter that you should pass on to your relatives so that they can better understand the result and pursue the proper care and testing. Please let them know that they can reach out to us at any time if they have questions or need help getting tested. This family letter serves as your chance to help the health of your family. If you would like to have an electronic copy of the letter, please email us at clinicalgenomics@geisinger.edu.

21. Lastly, so that I know how many family letters to send to you, do you mind if I ask if you have any siblings? Children? Are your parents still living? [Record how many 1st degree relatives]

© 2024 Schwartz MLB et al. *JAMA Network Open.*

22. **End with excellence:** I know that we just went over a lot of information and learning about this result can be surprising and upsetting for some– do you have any questions for me at this time? [If the patient seems distressed or has many questions- consider offering rapid appointment or discussion with on-call GC]

23. *Please keep in mind the next important steps: make an appointment to discuss this result and gather more information about your family history.* We will contact you in about one month to see how you are doing, check in about these important steps and see if you have any questions.

    a. If the patient requested the online tool for the cascade chatbot say: **We also have the online tool available for the one-month check in. Can we send you that on your computer, tablet or mobile phone as well?**

    But in the meantime, if you have questions or concerns, please call me at **570-714-6711**

© 2024 Schwartz MLB et al. *JAMA Network Open.*

eAppendix 3. Patient Information Packet Example Documents Used During Study Time Frame (Cover letter, scheduling information, and summary of genomic finding. Note: a family letter and copy of their genetic test report were also included in the packet but are not shown in the supplemental materials.)

DATE
PT NAME
PT ADDRESS

Dear Pt Name,

Thank you for being in the MyCode study at Geisinger. Samples from participants like you are being studied to help us learn more about human health and disease. Some participants will learn information important for *their* healthcare from MyCode.

We found medically important information in *your* sample. A change in your ________ gene may put you and some of your family members at risk for a type of _________ {condition description, e.g., 'heart muscle problem that can lead to abnormal heart rhythms', 'cancers like breast, ovarian, prostate, pancreatic, and melanoma', reaction to general anesthesia drugs and other muscle problems that could be triggered by heat or some medications }**. The name of the genetic condition related to this risk is** ________________ {condition name, e.g., "cardiomyopathy (CM)", "hereditary breast and ovarian cancer", "malignant hyperthermia"}**.**

{If participant not reached for disclosure}
We have made several efforts to review this result with you, including:

- __ MyGeisinger (Patient Portal) message: DATE(S)
- __ Phone call: DATE(S)

This letter marks our last try to contact you.

We recommend that you discuss this result with a genetic counselor and your family doctor. This result may be important for your health care now and in the future. **You should also share this result with your family members.** They may also have the same gene change and health risks. **Your first visit with a genetic counselor at Geisinger is free of charge**. Future medical appointments or testing you have because of this result are considered routine care. These future visits and tests will be billed to you or your health insurance.

Since you received a result, **you are included in a research registry.** This registry will follow your health related to your genetic test result over time. The registry collects information from your medical record. Occasionally you may be contacted to participate in surveys. If you do not want to be in this registry, please contact us at the number below. Choosing not to participate will NOT affect your care at Geisinger in any way.

We would be happy to review this result with you. We can also refer you to someone outside of Geisinger who can help. Please call (toll-free) **1-844-250-8031** or e-mail **MyCodeResults@geisinger.edu**.

The following is included in this mailing:

- A copy of your genetic test result for your records
- A letter that you can use to share your result with your family
- A fact sheet with information and suggested resources for people with these results

A copy of this letter and the genetic test result will be placed in your Geisinger electronic health record.

For questions or concerns about MyCode, please call (toll-free) **1-844-250-8031.** Thank you for your participation in MyCode and for trusting Geisinger with your care.

Sincerely,
The Genomic Counseling and Screening Team

For questions about your rights as a study participant, call the Geisinger Institutional Review Board. They can be reached at 570-271-8663. The study number is 2006-0258.

© 2024 Schwartz MLB et al. *JAMA Network Open.*

SCHEDULING INFORMATION

Geisinger has clinical genomics services available to individuals who have received DNA results from whole exome or genome sequencing and their family members. Clinical genomics providers can help you understand what a genetic test result means for the health of you and your family and the next steps for your medical care.

A typical clinical genomics visit includes talking about personal and family health history, the role of genes in health, and steps for managing and preventing disease. Some visits also include a diagnostic or physical examination. Appointments may include meeting with a Medical Geneticist, Genetic Counselor, Nurse Practitioner, or Physician's Assistant.

Services offered at each location: Below you will find scheduling location options that are available to you in addition to your primary care physician or outside provider. Each location offers the same clinical genomics services in order to help you better understand your result and how it affects you and your family. We offer in-person, telemedicine and telephone consults. Additional locations are becoming available as our practice expands.

To schedule an appointment at either location below, please call our clinic toll-free number, **1-844-250-8031**, anytime Monday-Friday 8:00 AM- 4:30 PM.

GEISINGER CLINICAL GENOMICS LOCATIONS

(A) Precision Health Center
(Forty-Fort, PA)
(B) Geisinger Medical Center
(Danville, PA)
(C) Gray's Woods
(State College)
(D) Scenery Park
(State College)
(E) Lewistown Medical Clinic
(Lewistown, PA)
(F) GENOMICS TELEMEDICINE/TELEPHONE (VARIOUS LOCATIONS)
Genomics telemedicine conferences or telephone consultations are also available for patients who are not able to travel.

What is a telemedicine conference?

Telemedicine is a live audio/video consult so patients can see and speak directly to a specialist in real time. Both sides have the ability to see reports, lab results, etc.

What is a telephone consultation?

A telephone consult is simply a phone call with a specialist regarding the details of your results and to discuss the resources available to you. This may be a convenient option for those who live far away and cannot travel to our on-site locations.

If your insurance requires a referral to see a specialist:
Geisinger providers can place a referral to our clinic in EPIC by selecting "Medical Genomics Referral OP [EPIC7437]." Non-Geisinger providers can fax referrals to Geisinger Clinical Genomics,
Fax Number 570-714-6601.

© 2024 Schwartz MLB et al. *JAMA Network Open.*

Summary of Genetic Finding: *MYBPC3* Pathogenic Variant
Risk for Cardiomyopathy

Why is this important medical information?

- This variant (change) means that one copy of your *MYBPC3* gene does not work properly.
- *MYBPC3* gene changes can cause a heart muscle disease known as cardiomyopathy. Cardiomyopathies are diseases of the heart muscle that make it harder for the heart to pump blood throughout the body.
- There are things you can do to lower your risk for cardiomyopathy.
- It is important to talk to your doctor about this genetic finding to guide your treatment.

What are the health risks for Cardiomyopathy?

- There are different types of heart muscle changes in different cardiomyopathies. These include:
  - Hypertrophic cardiomyopathy is a thickening of the heart muscle.
  - Dilated cardiomyopathy is a widening of one or more heart chambers.
  - Left Ventricular Noncompaction occurs when the lower left ventricle of the heart does not form correctly.
- People who have cardiomyopathy are at increased risk for heart failure and heart rhythm abnormalities (arrhythmias) which can lead to fainting, stroke or sudden cardiac arrest (the heart stops beating).
- Common symptoms for individuals with cardiomyopathy can include tiredness, shortness of breath, and fluid build-up in your arms or legs.

Specific Issues to Discuss with your Doctor

What are the next steps for my care?

- Talk to your healthcare provider about these results to personalize your treatment plan. This plan may include:
  - Ongoing imaging of the heart including an Echocardiogram or Cardiac MRI (magnetic resonance imaging).
  - Ongoing electrical studies of the heart including an Electrocardiogram (ECG or EKG) and Holter monitor.
  - Medications to help treat symptoms associated with a cardiomyopathy or reduce risk for cardiomyopathy.
  - Consider surgical options for people with an arrhythmia or heart failure.
- Pregnancy and Cardiomyopathy
  - If planning a pregnancy or currently pregnant, it is very important to discuss this genetic finding with your healthcare provider for medical management.
  - Pregnancy can increase risk for cardiomyopathy. This is called peripartum or pregnancy associated cardiomyopathy.
  - Pregnancy may be cautioned for some women who have advanced heart failure symptoms or cardiomyopathy.

Lifestyle changes

Avoidance of:

- Endurance training

© 2024 Schwartz MLB et al. *JAMA Network Open.*

- High intensity sports
- Dehydration
- Drinking too much alcohol
- Certain medications
- Using jacuzzi, steam room, or sauna

Additional medical specialists that may be relevant
You may be referred to see multiple cardiac specialists including a Cardiologist, Electrophysiologist, and Cardiothoracic surgeon for ongoing management and surveillance. Your doctor may also recommend a medical geneticist and/or genetic counseling visit.

How this Might Affect Family Members

How is this passed on in families?
- Genes are passed down in families from parent to child. Every person has two copies of the *MYBPC3* gene, one from their mother and one from their father. One changed copy of the gene is enough to cause risk for Cardiomyopathy even if the matching copy is working correctly.
- Within a family, the same gene changes can sometimes result in no symptoms, different symptoms, and/or symptoms at different ages.

Could my children, brothers, sisters, or other family members also have this condition?
- Each one of your children, parents, and full siblings has a 50% (1 in 2) chance to also have this genetic variant. A "full sibling" is a brother or sister who has the same mother and same father as you.
- Other relatives like aunts, uncles, cousins, and grandchildren could also have a changed copy of *MYBPC3* gene.

Genetic testing for family members
- We strongly encourage you to share this information with your blood relatives. Family sharing letters are provided in this packet to help you share this information with relatives.
- Genetic testing can clarify who has inherited the genetic risk for Cardiomyopathy and who has not.
- Family members related to you by blood can have genetic testing for the specific gene change identified in you and can use results to guide their care. This testing is usually done on a blood or saliva sample.
- The Geisinger Clinical Genomics Team can help your family members get the correct genetic testing or find a local genetics provider. They can be reached at **1-844-250-8031**.
- We do not recommend using MyCode as a way to have this type of genetic testing. The research project is not designed to provide results to family members quickly.

My relative does not want to have genetic testing – what now?
If a family member does *not* want to have genetic testing, they should still speak with their doctor about increased screening for Cardiomyopathy.

Information about the *MYBPC3* Gene

What does the *MYBPC3* gene do?
The heart is made up of muscle cells. The *MYBPC3* gene provides directions for making an important protein in the muscle cell. The *MYBPC3* protein keeps the heart muscle cells from breaking down. It also helps to keep the rate of muscle contraction, or “squeeze”, of the heart normal.

How do changes in the *MYBPC3* gene cause health problems?
Some variants in this gene cause disease by affecting the heart muscle. They may cause one of the chambers of the heart to change size or shape. This can affect pumping of blood.

Cardiomyopathy Resources

1. **American Heart Association:** A national organization with a mission to build healthier lives, free of cardiovascular disease. www.heart.org

2. **The Children's Cardiomyopathy Foundation (CCF):** The vision of the Children's Cardiomyopathy Foundation (CCF) is to create a future of hope in which pediatric cardiomyopathy can be detected earlier and any affected child can be cured to live a full and active life. http://www.childrenscardiomyopathy.org/

3. **The Cardiomyopathy UK organization:** A charity in the United Kingdom helping people with cardiomyopathy lead long and fulfilling lives. They provide information and support to families and help to train doctors and nurses. http://www.cardiomyopathy.org/

4. **Genetics Home Reference, MYBPC3 Gene:** This is a patient-friendly resource about the LMNA gene created by the U.S. National Library of Medicine. https://ghr.nlm.nih.gov/gene/MYBPC3

General Resources

1. **GenomeConnect**: A online patient registry that helps participants find others with similar DNA changes and relevant research studies. It is funded by the National Institutes of Health. www.genomeconnect.org

2. **Clinical Trials:** If you are interested in taking part in research, visit this site once or twice a year to see if you qualify for any research studies. http://clinicaltrials.gov

3. **Geisinger MyCode:** Information about Geisinger's MyCode Community Health Initiative. www.geisinger.org/mycode

4. **The National Society of Genetic Counselors “Find a Genetic Counselor tool”:** The website of the national foundation can help you find a genetic counselor in a specific geographic area. www.findageneticcounselor.com

Have Questions?
Please contact the MyCode Genomic Screening and Counseling team
with any questions about your DNA result.
1-844-250-8031
MyCodeResults@geisinger.edu

© 2024 Schwartz MLB et al. *JAMA Network Open.*

Summary of Genetic Finding: *BRCA2* Pathogenic Variant
Risk for Hereditary Breast and Ovarian Cancer (HBOC)

Why is this important medical information?

- This variant (change) in the *BRCA2* gene is associated with a condition called Hereditary Breast and Ovarian Cancer Syndrome, or HBOC. HBOC puts a person at an increased risk of developing certain cancers that run in a family.
- The following cancers are associated with *BRCA2* gene changes: breast, ovary, prostate, male breast, pancreas, and melanoma. Cancer screening for women begins at age 25, and for men at age 35. Childhood cancers are ***not*** known to be associated with HBOC.
- With appropriate screening and management, many of these cancers can be prevented or detected at an early, treatable, stage. Knowledge of these higher cancer risks will help you and your doctors to develop a plan that is right for you.
- It is important to talk to your doctor about this genetic finding.

What are the cancer risks for HBOC?

The table below shows the risks of developing cancer associated with HBOC. These risks are over a lifetime and are compared with the risk of developing cancer for someone in the general population (someone who does not have a *BRCA2* variant).

Cancer Risk to Age 70 Years in Individuals with a BRCA2 Variant Compared to the General Population		
Cancer	Risk in the General Population	Risk with a BRCA2 Variant
Female Breast	12%	45%
Male Breast	0.1%	5-10%
Ovarian	1.3%	11-17%
Pancreatic	1.5%	2-7%
Prostate	15%	<39%
Melanoma	2%	5%

It is possible to have HBOC and ***never*** develop cancer because there is ***not*** a 100% risk for any of the cancers listed above.

Specific Issues to Discuss with your Doctor

What are the next steps for my care?

The tables below show the current National Comprehensive Cancer Network (NCCN) guidelines for cancer screening and prevention for women and men. These guidelines change over time, so it is important to continue to talk with your doctors about your cancer screening options.

Screening and Prevention Options for Women with HBOC			
Type	Age to Begin	When to Repeat	Other Information
Breast Self-Awareness	18	Regularly	Know what is normal for you, and report changes to your doctor

© 2024 Schwartz MLB et al. *JAMA Network Open.*

**Clinical Breast Exam**	25	Every 6-12 months	Your doctor can help you recognize important changes in your breast
**Breast MRI**	25	Yearly	Mammogram if no breast MRI available
**Mammogram**	30	Yearly	Continue yearly breast MRI in addition to mammogram
**Surgery to remove ovaries**	35-40 and done having kids	N/A	Significantly reduces risk of ovarian and breast cancers
**Optional: Medications to reduce risk of breast cancer**	Talk with your doctor	N/A	Can significantly reduce the risk of breast cancer
**Optional: Surgery to remove breast tissue**	Talk with your doctor	N/A	Significantly reduces the risk of breast cancer
**Screening and Prevention Options for Men with HBOC**			
**Breast Self-Awareness**	35	Regularly	Know what is normal for you, and report changes to your doctor
**Clinical Breast Exam**	35	Yearly	Your doctor can help you recognize important changes in your breast
**Prostate Screening**	45	Yearly	Talk to your doctor

Lifestyle changes
Sun protection, such as wearing sun screen and wide brimmed hats, can minimize the risk of melanoma. Talk to your doctor about a full body skin and eye exam if you have a family history of melanoma.

Additional medical specialists that may be relevant
It is important for anyone who has a BRCA2 variant to follow specific early cancer screening and prevention guidelines.

For Women:

- There are doctors throughout the Geisinger system who specialize in helping people with *BRCA1* variants to develop a screening and management plan that is right for them. Consider talking about this result with:
- Your primary care provider will continue to manage your overall care.
- Geisinger Inherited Risk Breast Clinic
  - A clinic designed especially for people with *BRCA1* variants to meet with four HBOC specialists on the same afternoon. https://www.youtube.com/watch?v=_bkh3MqOFPE
  - If you have any questions about the clinic, you can call our cancer genetics department at 570-214-2637 and ask to speak to a genetic counselor about the Inherited Risk Breast Clinic.
- Gynecologic Oncologist or Gynecologist
  - A gynecologic oncologist can discuss removal of the ovaries (after age 35 *and* when you are done having kids)

For Men:

© 2024 Schwartz MLB et al. *JAMA Network Open.*

- There are doctors throughout the Geisinger system who specialize in helping people with BRCA1 variants to develop a screening and management plan that is right for them. Consider talking about this result with:
    - Your primary care provider will continue to manage your overall care and cancer screening.
    - Consider meeting with a Urologic Oncologist to monitor prostate cancer risks.

How this Might Affect Family Members

How is this passed on in families?

- Genes are passed down in families from parent to child. Every person has two copies of the *BRCA2* gene, one from their mother and one from their father. One broken copy of the gene is enough to cause risk for HBOC even if the matching copy is working correctly.
- Anyone with a broken copy of the gene has a 50% (1 in 2) chance to pass it on to each of their children.
- People who do not have a broken copy of the gene cannot pass it on to their children.
- Within a family, the same gene changes can sometimes result in no cancer, different cancer diagnosis, and/or cancer diagnoses at different ages.

Could my children, siblings, or other family members also have this condition?

- Each one of your children, parents, and full siblings has a 50% (1 in 2) chance to also have this broken copy of the *BRCA2* gene. A “full sibling” is a brother or sister who has the same mother and same father as you.
- Other relatives like aunts, uncles, cousins, and grandchildren could also have a broken copy of *BRCA2* gene.

Genetic testing for family members

- <u>We strongly encourage you to share this information with your blood relatives.</u> Family sharing letters are provided in this packet to help you share this information with relatives.
- Genetic testing can clarify who has inherited the genetic risk for cancer and who has not.
- Family members related to you by blood can have genetic testing for the specific gene change identified in you and can use results to guide their care. This testing is usually done on a blood or saliva sample.

- The Geisinger Clinical Genomics Team can help your family members get the correct genetic testing or find a local genetics provider. They can be reached **at 1-844-250-8031**.
- We <u>do not</u> recommend using MyCode as a way to have this type of genetic testing. The research project is not designed to provide results to family members quickly.

My relative does not want to have genetic testing – what now?

If a family member does *not* want to have genetic testing, they should still speak with their doctor about increased screening for *BRCA2* related cancers.

Information about the *BRCA2* Gene

What does the *BRCA2* gene do?

The *BRCA2* gene is a “tumor suppressor gene” – it typically stops cells from growing out of control.

© 2024 Schwartz MLB et al. *JAMA Network Open.*

How do changes in the *BRCA2* gene cause health problems
When the *BRCA2* gene is not working, cells in certain parts of the body are more likely to grow out of control. This causes an increased risk to develop certain types of cancer. However, with appropriate screening and management, many of these cancers can be prevented or detected at an early, treatable, stage.

***BRCA2* Resources**

1. **FORCE: Facing Our Risk of Cancer Empowered:** A patient-centered non-profit foundation that supports research, advocacy, and education about HBOC. www.facingourrisk.org Phone: 1-866-288-7475.
    a. Local FORCE outreach volunteer, Krystle Goverick, can be reached at krystleg@facingourrisk.org or 570-490-9100. Contact Krystle to help identify local resources, in-person support groups, and one-to-one support.

2. **Bright Pink:** A patient-centered non-profit organization that focuses on support and resources for young women at increased risk of breast and ovarian cancers. www.brightpink.org Phone: 312-787-4412

3. **Genetics Home Reference, *BRCA2* Gene:** A patient-friendly resource about the *BRCA2* gene created by the U.S. National Library of Medicine.https://ghr.nlm.nih.gov/gene/BRCA2

General Resources

1. **GenomeConnect:** A online patient registry that helps participants find others with similar DNA changes and relevant research studies. It is funded by the National Institutes of Health. www.genomeconnect.org

2. **Clinical Trials:** If you are interested in taking part in research, visit this site once or twice a year to see if you qualify for any research studies http://clinicaltrials.gov

3. **Geisinger MyCode:** Information about Geisinger's MyCode Community Health Initiative https://www.geisinger.org/mycode

4. **The National Society of Genetic Counselors "Find a Genetic Counselor tool":** The web site of the national foundation can help you find a genetic counselor in a specific geographic area. http://www.nsgc.org/page/find-a-gc-search

Have Questions?
Please contact the MyCode Genomic Screening and Counseling team
with any questions about your DNA result.
1-844-250-8031
MyCodeResults@geisinger.edu

© 2024 Schwartz MLB et al. *JAMA Network Open.*

Summary of Genetic Finding: *RYR1* Likely Pathogenic Variant
Risk for Malignant Hyperthermia Susceptibility

Why is this important medical information?

- This variant (change) in the *RYR1* gene causes a condition known as Malignant Hyperthermia (MH) Susceptibility.
- People with MH Susceptibility are at increased risk for a life-threatening reaction to certain medications used to put people to sleep for medical procedures (anesthesia).
- It is important to talk to your doctor about this genetic finding because <u>malignant hyperthermia reactions can be prevented and treated if your doctors know you are at risk.</u>

What are the risks for Malignant Hyperthermia (MH) Susceptibility?

- Malignant hyperthermia is a severe reaction that happens shortly after a person is given certain anesthesia medications.
    - When someone is having an MH reaction, their muscles contract too much. This can cause muscle breakdown, a high fever, extra acid in the body, a fast heartbeat, and kidney failure. An MH reaction can lead to death without treatment.
    - Some people with *RYR1* gene changes may have less severe symptoms after being given certain anesthesia medications such as muscle pain and dark urine due to muscle breakdown.
- People with *RYR1* variants can still be at risk for an MH event <u>even if they have not had problems when receiving anesthesia in the past.</u>
- Some people at risk for MH may also develop symptoms like heat stroke after being in hot environments or after extreme exercise.
- In addition to MH Susceptibility, certain *RYR1* variants can also cause lifelong problems with muscle weakness.

Specific Issues to Discuss with your Doctor

What are the next steps for my care?

- There are safe medicines that can be used if you need to be put to sleep for a medical procedure or surgery. Doctors can avoid giving you anesthesia medications that can cause a reaction if they know you have MH Susceptibility. You should tell your healthcare providers about your risk for MH.
- If you do have an MH reaction, there is a drug that can be used to treat you. This drug (Dantrolene sodium) is required to be in every operating room in the United States.
- You should avoid extremely hot temperatures and extreme physical exertion. There is no need to restrict routine exercise unless you are having signs of muscle breakdown or a heat stroke.

Lifestyle changes

- You should wear a medical ID to let doctors know you are at risk for MH. A medical ID is usually an easily recognized bracelet or necklace pendant. This can help if need to be put to sleep but cannot communicate about your MH risk such as after a car accident.
    - Medic alert forms to apply for an ID can be found in healthcare and pharmacy locations or on the internet at https://www.medicalert.org/.

Additional medical specialists that may be relevant

© 2024 Schwartz MLB et al. *JAMA Network Open.*

- You may be referred to a doctor with expertise in MH Susceptibility (often an anesthesiologist or nurse anesthetist).
- Your doctor may also recommend a medical genetics and/or genetic counseling visit.

How this Might Affect Family Members

How is this passed on in families?

- Genes are passed down in families from parent to child. Every person has two copies of the *RYR1* gene, one from their mother and one from their father. The gene change found in you causes the gene to not work properly.
- One *RYR1* gene change is enough to put a person at risk for an MH reaction even if the second copy is working correctly.
- Anyone with a *RYR1* gene change has 50% (1 in 2) chance to pass it on to each of their children.
- Within a family, the same gene change can sometimes result in no symptoms, different symptoms, and/or symptoms at different ages.

Could my children, brothers, sisters, or other family members also have this condition?

- Each one of your children, parents, and full siblings has a 50% (1 in 2) chance to also have this gene change. A "full sibling" is a brother or sister who has the same mother and same father as you.
- Other relatives like aunts, uncles, cousins, and grandchildren could also have this gene change.

Genetic testing for family members

- We strongly encourage you to share this information with your blood relatives. Family sharing letters are provided in this packet to help you share this information with relatives.
- Genetic testing can clarify who has inherited the genetic risk for MH and who has not.
- Family members related to you by blood can have genetic testing for the specific gene change identified in you and can use results to guide their care. This testing is usually done on a blood or saliva sample.
- The Geisinger Clinical Genomics Team can help your family members get the correct genetic testing or find a local genetics provider. They can be reached **at 1-844-250-8031**.
- We do not recommend using MyCode as a way to have this type of genetic testing. The research project is not designed to provide results to family members quickly.

My relative does not want to have genetic testing – what now?

If a family member does *not* want to have genetic testing, they should still notify their healthcare providers that they are at risk for an MH reaction.

Information about the *RYR1* Gene

What does the *RYR1* gene do?

The *RYR1* gene provides the instructions for making a protein which controls how calcium moves in and out of muscle cells. Calcium in our muscles helps make them move.

© 2024 Schwartz MLB et al. *JAMA Network Open.*

How do changes in the *RYR1* gene cause health problems
RYR1 gene changes affect how the *RYR1* gene works in the body. These gene changes cause the *RYR1* protein to work differently in response to certain anesthesia medications. This can change how calcium moves in the muscles and cause an MH reaction.

***RYR1* Resources**

1. **Malignant Hyperthermia Association of the United States (MHAUS):** MHAUS promotes care and understanding of MH and similar conditions. They have many resources including a list of MH event triggering medications, a patient registry and other informational materials. http://www.mhaus.org/patients-and-families

2. **MedicAlert Foundation:** The MedicAlert Foundation can work with you to get a Medical ID. They also provide other services like 24/7 emergency responses, family notification, and emergency wallet cards. https://www.medicalert.org/

3. **Genetics Home Reference, *RYR1* Gene**: This is a patient-friendly resource about the RYR1 gene created by the U.S. National Library of Medicine. https://ghr.nlm.nih.gov/gene/RYR1

General Resources

1. **GenomeConnect:** A online patient registry that helps participants find others with similar DNA changes and relevant research studies. It is funded by the National Institutes of Health. www.genomeconnect.org

2. **Clinical Trials:** If you are interested in taking part in research, visit this site once or twice a year to see if you qualify for any research studies http://clinicaltrials.gov

3. **Geisinger MyCode:** Information about Geisinger's MyCode Community Health Initiative https://www.geisinger.org/mycode

4. **The National Society of Genetic Counselors "Find a Genetic Counselor tool":** The web site of the national foundation can help you find a genetic counselor in a specific geographic area. http://www.nsgc.org/page/find-a-gc-search

Have Questions?
Please contact the MyCode Genomic Screening and Counseling team
with any questions about your DNA result.
1-844-250-8031
MyCodeResults@geisinger.edu

© 2024 Schwartz MLB et al. *JAMA Network Open.*

eTable 1. Dummy Coding for Categorical Variables

Variable	Dummy coding	Note
Age at result disclosure in years	18-40<br>41-65<br>66-80<br>81+ (reference)	
Charlson comorbidity index	0–2<br>3–4<br>5+ (reference)	
Distance to closest genetic counseling clinic	Distance <= 8.9<br>8.9-13.3<br>13.3-20.1<br>>20.1 (reference)	By quantiles: q1, q2, q3
Time from results disclosure to data collection	Time<2year (reference)<br>>2year<br>>3year<br>>4year<br>>5year	By 1-year interval

© 2024 Schwartz MLB et al. *JAMA Network Open.*

eTable 2. Variable Selection Before and After Backward Elimination

All predictors	After backward elimination
Age at result disclosure[a]	Age at result disclosure[a]
Sex	Sex
Race	Marital status[a]
Marital status[a]	Comorbidity index[a]
Comorbidity index	Patient portal user
Primary care physician status	Gene category[a]
Patient portal user	Disclosure staff type[a]
Gene category[a]	Distance to closest genetic counseling clinic[a,b]
Disclosure staff type[a]	
Employment status[a]	
Distance to closest genetic counseling clinic (miles)[a]	
Time from results disclosure to data collection (days)[a]	

[a]use dummy coding in logistic regression model; [b]categorized by quantiles (q1, q2, q3)

© 2024 Schwartz MLB et al. *JAMA Network Open.*

eTable 3: Number of Participants With Results by Gene

Gene	Condition	Condition Category	Participants[a]
ACTA2	Hereditary thoracic aortic disease	Cardiovascular	17
APC	Familial adenomatous polyposis	Cancer	13
APOB	Familial hypercholesterolemia	Cardiovascular	35
BRCA1	Hereditary breast and ovarian cancer syndrome	Cancer	97
BRCA2	Hereditary breast and ovarian cancer syndrome	Cancer	218
COL3A1	Vascular Ehlers-Danlos syndrome	Cardiovascular	6
DSC2	Arrhythmogenic cardiomyopathy	Cardiovascular	8
DSG2	Arrhythmogenic cardiomyopathy	Cardiovascular	22
DSP	Arrhythmogenic cardiomyopathy	Cardiovascular	34
ENG	Hereditary hemorrhagic telangiectasia	Cardiovascular	1
FBN1	Marfan syndrome	Cardiovascular	11
GLA	Fabry disease	Cardiovascular	2
KCNE1	Long QT syndrome	Cardiovascular	3
KCNH2	Long QT syndrome	Cardiovascular	9
KCNQ1	Long QT syndrome	Cardiovascular	73
LDLR	Familial hypercholesterolemia	Cardiovascular	117
LMNA	Dilated/hypertrophic cardiomyopathy	Cardiovascular	6
MEN1	Multiple endocrine neoplasia type 1	Cancer	3
MLH1	Lynch syndrome	Cancer	9
MSH2	Lynch syndrome	Cancer	8
MSH6	Lynch syndrome	Cancer	71
MYBPC3	Dilated/hypertrophic cardiomyopathy	Cardiovascular	52
MYH7	Dilated/hypertrophic cardiomyopathy	Cardiovascular	24
MYL2	Dilated/hypertrophic cardiomyopathy	Cardiovascular	3
MYL3	Dilated/hypertrophic cardiomyopathy	Cardiovascular	4
PKP2	Arrhythmogenic cardiomyopathy	Cardiovascular	23
PMS2	Lynch syndrome	Cancer	70
PTEN	*PTEN* hamartoma tumor syndrome	Cancer	5
RB1	Hereditary retinoblastoma	Cancer	1
RET	Multiple endocrine neoplasia type 2	Cancer	29
RYR1	Malignant hyperthermia	Other	84
SCN5A	Brugada syndrome	Cardiovascular	44
SDHAF2	Hereditary paraganglioma-pheochromocytoma syndrome	Cancer	2
SDHB	Hereditary paraganglioma-pheochromocytoma syndrome	Cancer	15

© 2024 Schwartz MLB et al. *JAMA Network Open.*

SDHC	Hereditary paraganglioma-pheochromocytoma syndrome	Cancer	9
SDHD	Hereditary paraganglioma-pheochromocytoma syndrome	Cancer	5
SMAD3	Loeys-Dietz syndrome	Cardiovascular	1
TGFBR1	Loeys-Dietz syndrome	Cardiovascular	1
TNNI3	Dilated/hypertrophic cardiomyopathy	Cardiovascular	4
TNNT2	Dilated/hypertrophic cardiomyopathy	Cardiovascular	6
TP53	Li-Fraumeni syndrome	Cancer	12
TPM1	Dilated/hypertrophic cardiomyopathy	Cardiovascular	2
TSC1	Tuberous sclerosis complex	Cancer	3
TSC2	Tuberous sclerosis complex	Cancer	3
VHL	Von Hippel-Lindau syndrome	Cancer	2
ACTC1	Dilated/hypertrophic cardiomyopathy	Cardiovascular	0
ACVRL1	Hereditary hemorrhagic telangiectasia	Cardiovascular	0
ATP7B	Wilson disease	Other	0
BMPR1A	Juvenile polyposis	Cancer	0
CACNA1S	Malignant hyperthermia	Other	0
MUTYH	*MUTYH*-associated polyposis	Cancer	0
MYH11	Hereditary thoracic aortic disease	Cardiovascular	0
MYLK	Hereditary thoracic aortic disease	Cardiovascular	0
NF2	Neurofibromatosis type 2	Cancer	0
OTC	Ornithine transcarbamylase deficiency	Other	0
PCSK9	Familial hypercholesterolemia	Cardiovascular	0
PRKAG2	Dilated/hypertrophic cardiomyopathy	Cardiovascular	0
RYR2	Catecholaminergic polymorphic ventricular tachycardia	Cardiovascular	0
STK11	Peutz-Jeghers syndrome	Cancer	0
TGFBR2	Loeys-Dietz syndrome	Cardiovascular	0
TMEM43	Arrhythmogenic cardiomyopathy	Cardiovascular	0
WT1	Wilms tumor	Cancer	0

[a]7 participants had two genetic results

© 2024 Schwartz MLB et al. *JAMA Network Open.*

eTable 4. Visit Completion (n, %) as Stratified by Follow-up Duration in Years Since Result Disclosure

Interval (year)	0-2	2-3	3-4	4-5	>5
Sample size	398	289	240	160	73
Completion, n (%)	167 (42%)	141 (49%)	119 (50%)	86 (54%)	38 (52%)

© 2024 Schwartz MLB et al. *JAMA Network Open.*

eTable 5. Association of Participant Characteristics With Visit Completion

Characteristic	OR (95% CI)	p-value
Age_18-40 (vs. 81+)	2.98 (1.40, 6.53)	0.005
Age_41-65 (vs. 81+)	2.36 (1.22, 4.74)	0.013
Age_66-80 (vs. 81+)	2.60 (1.41, 4.98)	0.003
Sex_Female (vs. Male)	1.49 (1.14, 1.96)	0.004
Marital_Married (vs. Single)	1.74 (1.23, 2.47)	0.002
Marital_Divorced (vs. Single)	1.80 (1.11, 2.91)	0.017
Marital_Widowed (vs. Single)	1.06 (0.59, 1.91)	0.835
CCI_0-2 (vs. 5+)	1.76 (1.16, 2.68)	0.008
CCI_3-4 (vs. 5+)	1.73 (1.18, 2.54)	0.005
Patient portal user (Yes vs. No)	1.42 (1.06, 1.89)	0.017
Gene_cancer (vs. other)	2.13 (1.28, 3.58)	0.004
Gene_CVD (vs. other)	1.60 (0.96, 2.70)	0.071
Discloser_GC (vs. not disclosed)	16.32 (8.16, 37.45)	<0.001
Discloser_RA (vs. not disclosed)	20.30 (10.25, 46.31)	<0.001
Distance 0-8.9 (vs. >20.1)	1.64 (1.14, 2.36)	0.007
Distance 8.9-13.3 (vs. >20.1)	1.28 (0.89, 1.82)	0.181
Distance 13.3-20.1 (vs. >20.1)	1.25 (0.86, 1.81)	0.247

CCI=Charlson comorbidity index; CVD=cardiovascular disease; GC=genetic counselor; RA=research assistant

© 2024 Schwartz MLB et al. *JAMA Network Open.*

eTable 6. Participant Characteristics Stratified by Genetics Visit Completion, Only Including Variables Selection for Logistic Regression

	Not completed	Completed visit	p-value
Total sample size	609	551	
Age category, n (%)			<0.001
18-40	121 (19.9)	138 (25.0)	
41-65	268 (44.0)	256 (46.5)	
66-80	154 (25.3)	140 (25.4)	
81+	66 (10.8)	17 (3.1)	
Sex, Male (%)	267 (43.8)	190 (34.5)	0.001
Race, White (%)	595 (97.7)	537 (97.5)	0.939
Marital status, n (%)			0.001
Married/Significant other	320 (52.5)	346 (62.8)	
Single	138 (22.7)	98 (17.8)	
Divorced/Separated	77 (12.6)	71 (12.9)	
Widowed	73 (12.0)	36 (6.5)	
Comorbidity index, n (%)			<0.001
0-2	284 (46.6)	306 (55.5)	
3-4	124 (20.4)	131 (23.8)	
5+	201 (33.0)	114 (20.7)	
Patient portal user, Yes (%)	392 (64.4)	415 (75.3)	<0.001
Gene category, n (%)			0.056
Cancer risk	283 (46.5)	289 (52.5)	
Cardiovascular disease risk	274 (45.0)	230 (41.7)	
Other disease risk	52 (8.5)	32 (5.8)	
Disclosure staff type, n (%)			<0.001
Genetic counselor	222 (36.5)	217 (39.4)	
Research assistant	269 (44.2)	326 (59.2)	
Not disclosed	118 (19.4)	8 (1.5)	
Distance to clinic (miles), n (%)			0.131
0-8.9	140 (23.0)	154 (27.9)	
8.9-13.3	161 (26.4)	151 (27.4)	
13.3-20.1	150 (24.6)	128 (23.2)	
>20.1	158 (25.9)	118 (21.4)	

© 2024 Schwartz MLB et al. *JAMA Network Open.*

eTable 7. Factors Significantly Associated With Completion of Genetics Visit Among Those Reached for Disclosure

	Category 1	Category 2 (reference)	OR (95% CI)	p-value
Age				
	18-40	81+	2.74 (1.28, 6.03)	0.010
	41-65	81+	2.38 (1.22, 4.79)	0.012
	66-80	81+	2.63 (1.42, 5.05)	0.003
Sex	Female	Male	1.48 (1.12, 1.95)	0.005
Marital status				
	Married/ Significant other	Single	1.73 (1.22, 2.47)	0.002
	Divorced	Single	1.86 (1.14, 3.05)	0.014
Comorbidity index				
	0-2	5+	1.79 (1.17, 2.75)	0.007
	3-4	5+	1.73 (1.17, 2.56)	0.006
Patient portal user	Yes	No	1.44 (1.08, 1.94)	0.014
Gene Category				
	Cancer	Other	2.06 (1.23, 3.46)	0.006
Distance to clinic (miles)				
	0-8.9	>20.1	1.64 (1.14, 2.37)	0.008

Note: The role of the person disclosing results (genetic counselor vs. research assistant) was not significantly associated with visit completion (p = 0.10).

© 2024 Schwartz MLB et al. *JAMA Network Open.*

eTable 8. Relationship Between 1-Month Follow-up Calls and Visit Completion Status Among Those who did not Schedule a Visit During an Initial Disclosure Attempt

**Visit completion status for fall 2019 patients that declined or deferred a visit on their disclosure call by one-month call status (N=141/398)**		
	Completed Visit	Did Not Complete Visit
Successful one-month Call	10	70
Unsuccessful one-month Call	6	55
OR = 1.30, 95% CI: [0.45, 3.83], p=0.64		
**Visit completion status among those who had not been reached at the time of a one-month follow-up call (N=90/398)**		
	Completed Visit	Did Not Complete Visit
Successful one-month Call	7	19
Unsuccessful one-month Call	6	58
OR = 3.56, 95% CI: [1.06, 11.91], p=0.047		

Visit completion status by one-month call status among fall 2019 patients that did not have a scheduled genetic counseling visit by one-month post-upload. 26 patients who were originally lost-to follow-up were reached for the first time on the one-month call

© 2024 Schwartz MLB et al. *JAMA Network Open.*
