# Supplementary material for: Genetics Visit Uptake Among Individuals Receiving Clinically Actionable Genomic Screening Results
Source: JAMA Netw Open. 2024 Mar 15;7(3):e242388. doi: 10.1001/jamanetworkopen.2024.2388 (PMC10943406; doi:10.1001/jamanetworkopen.2024.2388)
Supplement: Supplement 2. — Data Sharing Statement [file jamanetwopen-e242388-s002.pdf]

Data Sharing Statement

Schwartz. Genetics Visit Uptake Among Individuals Receiving Clinically Actionable Genomic Screening Results. *JAMA Netw Open*. Published March 14, 2024.
doi:10.1001/jamanetworkopen.2024.2388

Data
Data available: Yes
Data types: Deidentified participant data
How to access data: Adam Buchanan, ahbuchanan@geisinger.edu
When available: With publication

Supporting Documents
Document types: Informed consent form
How to access documents: Adam Buchanan, ahbuchanan@geisinger.edu
When available: With publication

Additional Information
Who can access the data: researchers whose proposed use of data has been approved
Types of analyses: for specified purpose
Mechanisms of data availability: signed data access agreement
